# Surviving Chytridiomycosis: Differential Anti-*Batrachochytrium dendrobatidis* Activity in Bacterial Isolates from Three Lowland Species of *Atelopus*

**DOI:** 10.1371/journal.pone.0044832

**Published:** 2012-09-10

**Authors:** Sandra V. Flechas, Carolina Sarmiento, Martha E. Cárdenas, Edgar M. Medina, Silvia Restrepo, Adolfo Amézquita

**Affiliations:** Department of Biological Sciences, Universidad de los Andes, Bogotá, Colombia; Smithsonian's National Zoological Park, United States of America

## Abstract

In the Neotropics, almost every species of the stream-dwelling harlequin toads (genus *Atelopus*) have experienced catastrophic declines. The persistence of lowland species of *Atelopus* could be explained by the lower growth rate of *Batrachochytrium dendrobatidis* (*Bd*) at temperatures above 25°C. We tested the complementary hypothesis that the toads' skin bacterial microbiota acts as a protective barrier against the pathogen, perhaps delaying or impeding the symptomatic phase of chytridiomycosis. We isolated 148 cultivable bacterial strains from three lowland *Atelopus* species and quantified the anti-*Bd* activity through antagonism assays. Twenty-six percent (38 strains representing 12 species) of the bacteria inhibited *Bd* growth and just two of them were shared among the toad species sampled in different localities. Interestingly, the strongest anti-*Bd* activity was measured in bacteria isolated from *A. elegans*, the only species that tested positive for the pathogen. The cutaneous bacterial microbiota is thus likely a fitness-enhancing trait that may (adaptation) or not (exaptation) have appeared because of natural selection mediated by chytridiomycosis. Our findings reveal bacterial strains for development of local probiotic treatments against chytridiomycosis and also shed light on the mechanisms behind the frog-bacteria-pathogen interaction.

## Introduction

The world's amphibians are facing a conservation crisis due to a variety of factors [1], with the newest and perhaps most insidious threat coming from an emerging global pathogen, the microscopic fungus *Batrachochytrium dendrobatidis*, or *Bd* [2]. This fungus is the etiological agent of chytridiomycosis, and has been implicated in many enigmatic declines worldwide [3], [4], [5]. In the Western Hemisphere, the disease has mainly affected populations in highland environments [6], [7], [8], [9]. Species occurring in tropical regions at middle and high elevations are thought to face a higher risk presumably because (1) *Bd* grows faster at the low temperatures characteristic of montane areas [10], [11], and (2) the amphibian immune capacity may decrease at lower temperatures [7], [12]. Despite the current focus on montane declines, a growing body of evidence suggests that *Bd* may also be widespread and abundant in lowland amphibian populations, for example, in Australia and Central America [11], [13], [14], yet amphibian declines are rarely observed in the lowlands [14], [15].

Individuals, populations and species of amphibians are known to vary in their susceptibility to chytridiomycosis, but the causes of this variation are not well understood [16]. Some species consist of both resistant and susceptible populations [6]. Some species maintain remnant populations that persist in the presence of *Bd* [17], [18], whereas others are known to be infected but show no signs of chytridiomycosis [5], [19]. The allegedly well developed immune system of amphibians, which shows both adaptive and innate responses [7], is probably involved on the resistance to infection [20], [21]. Amphibians may also benefit from symbiotic microbiota present on their skin that function as a barrier against pathogens, including *Bd*, which specifically targets amphibian skin [22], [23], [24]. Previous work has demonstrated that bacterial isolates from the skin of certain species of amphibians exhibit strong antifungal activity against *Mariannaea* sp. [25] and *Bd* [22], [26]. For example, the bacteria *Janthinobacterium lividum* and *Lysobacter gummosus*, produce the antimicrobial peptides violacein and 2,4-diacetylphloroglucinol respectively, which inhibit *Bd* growth *in vitro* [27], [28]. Thus, variation in the cutaneous microbial community of amphibian skin may be a key factor in resistance to chytridiomycosis [26], [28], [29], [30].

Conservation strategies focused on amphibians threatened by chytridiomycosis have mainly sought to prevent the spread of *Bd* to naïve populations and establish *ex situ* assurance colonies, yet solving the problem may require prophylactic treatments and enabling populations to persist with the pathogen [31]. Since the discovery of cutaneous bacteria with strong antifungal properties, increasing effort has focused on probiotic therapies [25], [30]. Perhaps the most promising approach to date is to use beneficial symbiotic microorganisms, or their metabolic products, to increase the resistance to infection or disease through environment bioaugmentation or host therapy [16], [30], [31], [32]. However, the majority of the studies had not considered the role of the environmental conditions on this host-pathogen interaction, since small variations could strongly affect anti-*Bd* activity of protective bacteria [33].

While most research on the immune-like properties of the cutaneous microbiota of amphibian skin has been conducted on temperate zone systems [32], [34], the vast majority of amphibian diversity lies in the tropical realm, especially in South America [35]. If microbial communities on tropical amphibians differ from those on temperate hosts, then probiotic therapies optimized for these highly endangered tropical species are needed [36]. Furthermore, since bioaugmentation and other probiotic approaches would eventually require the anthropogenic introduction of bacteria into an environment, using locally obtained bacteria might minimize the risks associated with this procedure. Therefore, surveying the diversity and evaluating the anti-*Bd* action of microbial communities in the amphibian skin of Neotropical species could potentially expand the array of tools to help mitigate the impact of *Bd*.

One of the more severely impacted groups of amphibians are the montane harlequin toads of the genus, *Atelopus* (Anura: Bufonidae) with 80% of species listed as critically endangered [37]. In Colombia nearly all the 33 species of *Atelopus* have declined sharply, yet four lowland species are persisting at 0–600 masl [38]. For at least one species, *A. elegans* from Gorgona Island, we know that the pathogen has been present for at least five years without causing obvious disease or declines [39]. Among the various possible factors that may account for the persistence of *A. elegans* despite infection with *Bd* we hypothesize that cutaneous symbiotic bacteria may be a contributing factor to disease resistance in this population.

The aim of our study was to test whether the frog *A. elegans* harbors cutaneous bacteria capable of inhibiting *Bd* growth. We also compared the antifungal bacterial communities found in *A. elegans* with two other *Atelopus* species that persist in the lowlands without evidence of *Bd* infection. Thus, our null hypothesis is that bacteria isolated from the infected species (*A. elegans*) should exhibit stronger anti-*Bd* activity compared with bacteria from the other two toad species. Our long-term goal is to know whether these potentially beneficial strains could be used in bioaugmentation experiments or the metabolites they produce employed in host therapy to protect threatened Neotropical species.

## Materials and Methods

### Ethics statement

Procedures for capture and handling of live animals in the field were approved by the Colombian National Parks authority and the Ministry of the Environment, under permits DTSO 019-09, DTSO 001-09 and N° 10-07032012.

### Study species

Cutaneous bacterial microbiota was sampled from three latitudinally separated species occurring in coastal forests of Colombia. *Atelopus* aff. *limosus*, probably an undescribed species, occurs near the municipality of Capurganá (8° 37′N, 77° 22′W, 150 masl), close to the border between Panama and Colombia; *Atelopus spurrelli*, considered as Vulnerable (VU) by the IUCN [40], was sampled near the municipality of Arusí (5°30′N, 77°31′W, 90 masl) on the Pacific coast; and *A. elegans*, Critically Endangered (CR), was sampled in the insular Gorgona National Park located 56 Km off the Pacific coast of Colombia (2°47′–3°6′ N, 78°6′–78°18′ W, between 6–115 masl). *A. elegans* had tested positive (17%, 15 out of 78 individuals) for *Bd* at the time we conducted this study [39]. We sampled 80 individuals of *A. spurrelli* and 82 of *A.* aff. *limosus* and detected no infected individuals. We thus concluded that *Bd* might be either absent or rare in those populations.

### Bacterial isolation

To obtain bacterial isolates from toads' skin, samples were collected from five *A. spurrelli*, eight *Atelopus* aff. *limosus* and seven *A. elegans* adults. Toads were manipulated using fresh disposable nitrile gloves and rinsed twice in sterile dechlorinated water to remove transient bacteria [25]. Individuals were swabbed on their left, right and ventral surfaces, hindlimbs and interdigital membranes, using a sterile cotton swab. Swabs were preserved in 2 mL cryovials containing 1 mL DS solution, a weak salt solution resembling pond water [41]. All swab samples were refrigerated within 24 h and processed within 48 hours after sampling. To isolate pure colonies, serial dilutions were performed until 1×10^−5^. To recover the highest possible number of bacterial morphotypes, each dilution was plated in R2A media in duplicate and incubated at 23°C for two days. Bacterial morphotypes were defined according to the macroscopic characteristics of the obtained colonies (i.e. color, form, elevation and margin). Single colonies of each bacterial morphotype were streaked on fresh nutritive agar plates until pure cultures were obtained. Each isolate was cryopreserved in nutritive broth with 30% glycerol at −80°C.

### Batrachochytrium dendrobatidis *growth inhibition assays*

To test for anti-*Bd* activity in bacteria isolated from frogs' skin, we used growth inhibition assays [22]. At the time of the assays, no Colombian *Bd* strains were available, so we decided to use *Bd* strain JEL 423 (University of Maine, Orono, USA) isolated from *Phyllomedusa lemur* in lowland forests of neighboring Panama. *Bd* was grown in TGh media (10 g tryptone, 10 g agar, 4 g gelatine hydrolisate, 1000 mL distilled water) for three days at 23°C until maximal zoospore production was observed. *Bd* was then harvested from three Petri dishes and transferred to a sterile tube containing 16 mL of sterile water in order to obtain a solution with a high concentration of zoospores. Plates with 25 mL of TGh media were coated with 1 mL of the zoospore suspension, and plates were allowed to dry until most of the solution had diffused into the agar. Unknown or “query” bacteria from active isolated cultures were streaked in a line across one side of a Petri dish. *Escherichia coli* (strain DH5α) was streaked as a negative control in a parallel line on the opposite side of the Petri dish; this control streak was included to observe the *Bd* growth around a bacterial species that shows no inhibition activity on *Bd* growth. Each bacterial isolate was tested in triplicate and the plates were incubated at 23°C. On the third day of incubation, query bacteria were checked for signs of inhibition of *Bd* growth. For those isolates showing inhibition, photographs were taken and anti-*Bd* activity was estimated by using photometric techniques.

**Figure 1:**
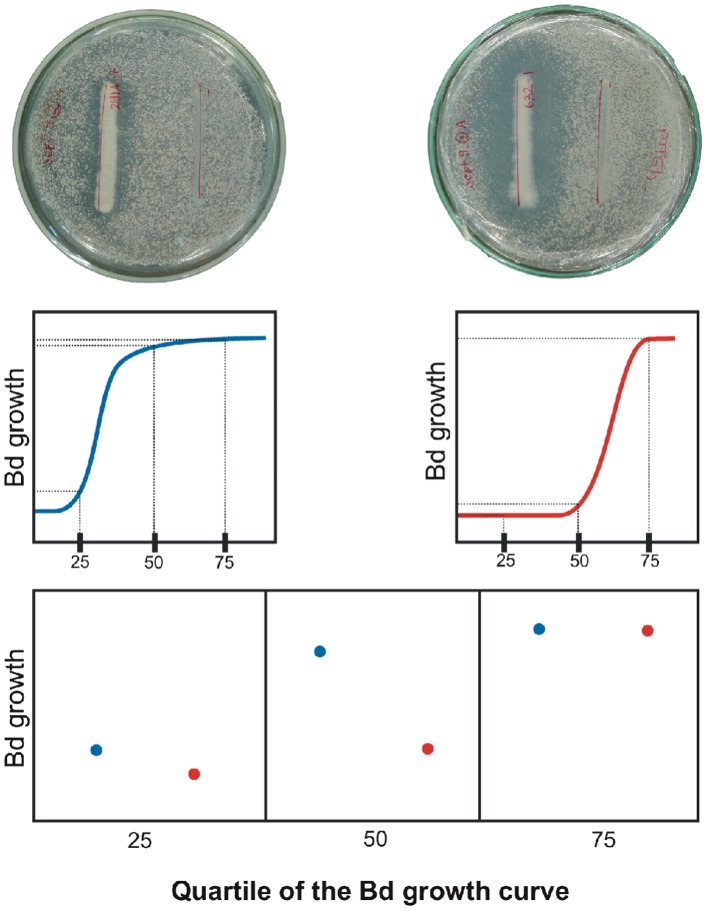
Photometric method. Estimation of bacterial anti-*Bd* effect as a combined measurement of the inhibition halo around the query bacteria (thick width band, above) and the decreasing density of *Bd* colonies. A spline regression model is fitted to grey values (i.e. density of *Bd* colonies) against distance to the query bacteria (middle). *Bd* growth is then interpolated at the first (25), second (50), and third (75) quartiles (middle and below). The bacterial isolate in the right column (red) have a stronger anti-*Bd* effect than the bacteria in the left column (blue).

We found two kinds of evidence of anti-*Bd* activity in the tested cultures. First, we detected an inhibition zone around the query bacteria line. Second, we found strong variation in the density of *Bd* colonies growing throughout the culture medium outside the inhibition zone. To summarize both effects in a single measurement we took photographs of the cultures under similar lighting conditions with a dark background. Then, we estimated *Bd* growth by measuring the light (i.e. grey value) reflected at variable distances of the query bacteria line (Fig. 1). Data from the three plates were averaged in order to obtain the statistical unit of analysis. To facilitate comparisons we re-expressed grey values as percentages of the maximum light intensity reflected by a single *Bd* colony within the same Petri dish. All photometric measurements were conducted on the software ImageJ [42] after distance and light calibration.

To quantify *Bd* growth inhibition we modeled Grey values (hereafter *Bd* growth) as a function of distance to the query bacteria by using spline models on JMP statistics software (JMP, Version 8. SAS Institute Inc., Cary, NC, 1989–2007). Spline models combine several polynomial functions of relatively low degree (often cubic) to fit piecewise several subsets of X–Y values. A Lambda parameter allows modifying the shape of the curve between a pure cubic function (Lambda  = 0) and eventually a straight line, as Lambda diverges to infinity [43]. The functions are blend smoothly allowing both interpolation of Y-values as well as estimation of model residuals.

The anti-*Bd* activity was compared between bacterial strains and frogs in two ways. First, we fitted a general spline model for all readings of *Bd* growth at every tested distance from all bacteria. The residuals of this model were then calculated by frog species and plotted to show between-frogs differences in the anti-*Bd* activity of their corresponding bacteria. Second, we fitted *Bd* growth against Distance to bacteria by using a single spline model for each bacterial strain. Because the Lambda value (**λ** = 1) and the range of Distance values (0.17–1.77 cm) were identical for every model, we could interpolate *Bd* growth values at the first, second and third quartile of the growth function and save them as new variables. Then, we tested the effect of frog species on the *Bd* growth quartiles by using multivariate analysis of variance. The first approach allowed easy visualization of the general pattern; the second one permitted statistical testing.

### Identification of bacterial isolates

Bacterial strains were identified by sequencing the 16S ribosomal gene. One colony of each morphotype was re-suspended in 10 μL of distilled water in a 0.2 mL PCR tube and boiled for 7 min at 95°C. The solution was used directly in a PCR reaction with the universal eubacterial primers, 27F (5′- AGAGTTTGATCCTGGCTCAG-3′) and 1492R (5′-GGTTACCTTGTTACGACTT-3′) [44]. Thermocycling parameters were: an initial denaturation of 3 min at 95°C, followed by 35 cycles of 45 s at 95°C, 45 s at 52°C and 90 s at 72°C. A final extension of 7 min at 72°C was performed to complete polymerization. PCR results were checked by electrophoresis in 1% agarose gels. Products were sent to Macrogen (Korea) for sequencing. DNA sequences were cleaned and assembled using Geneious [45]. 16S sequences were then identified using BLASTn [46] against the complete GenBank nucleotide database (http://www.ncbi.nlm.hih.gov) and the Greengenes database (http://greengenes.lbl.gov), using default parameter settings in both cases.

## Results

A total of 148 cultivable bacterial morphotypes were isolated, 40 from *Atelopus elegans*, 85 from *Atelopus* aff. *limosus*, and 23 from *A. spurrelli*. In antagonism assays, we observed anti-*Bd* activity in 16 of 40 (40%) bacterial morphotypes from *A. elegans*, 16 of 85 from *Atelopus* aff. *limosus* (19%), and six of 23 (26%) from *A. spurrelli*. We detected an inhibition zone around the query bacteria line, and also found strong variation in the density of *Bd* growing in the Petri dish outside the inhibition halo (Fig. 1).

Among all three hosts we identified 12 bacterial species with anti-*Bd* activity belonging to six genera: *Pseudomonas, Acinetobacter*, *Stenotrophomonas*, *Comamonas, Chryseobacterium* and *Elizabethkingia* (Table 1). Toad species differed more in the composition of bacteria with anti-*Bd* activity (Anosim R = 0.42, P<0.0001, 9999 permutations) than in the whole bacterial communities (R = 0.51, P<0.0001) as inferred as well from Bray-Curtiss indices of dissimilarity: 0.71 (0.59 for all cultivable bacteria) between *Atelopus* aff. *limosus* and *A. spurrelli*, 0.84 (0.69) between *Atelopus* aff. *limosus* and *A. elegans*, and 0.75 (0.71) between *A. spurrelli* and *A. elegans*, ranging between 0 =  identical and 1 =  totally dissimilar. Two out of the three strains that exhibited the highest anti-*Bd* action, both tentatively assigned to *P. tolaasii*, were exclusive to *A. elegans*. The third one, tentatively assigned to *P. putida*, was isolated from *A. spurrelli* skins.

**Table 1:** Prevalence of bacterial species isolated from the skin of *Atelopus* aff. *limosus, A. spurrelli* and *A. elegans*.

Bacterial Isolates	*Atelopus* aff. *limosus*	*Atelopus spurrelli*	*Atelopus elegans*
*Acinetobacter baumanii*	3	2	0
*Acinetobacter calcoaceticus*	2	1	0
*Acinetobacter genomosp.*	1	0	0
*Acinetobacter gyllenbergii*	5*	1*	0
*Acinetobacter venetianus*	1	0	0
*Acinetobacter haemolyticus*	0	1*	0
*Acinetobacter junii*	1	0	0
*Acinetobacter* sp.	7*	2*	3*
*Chryseobacterium* sp.	5*	0	3
*Comamonas* sp.	1	1	5*
*Comamonas testosteroni*	1	2	2
*Cupriavidus metallidurans*	0	0	1
*Elizabethkingia meningosepticum*	1*	1	0
*Pseudomonas* sp.	1	1	0
*Pseudomonas aeruginosa*	0	0	1*
*Pseudomonas putida*	1*	2*	1*
*Pseudomonas nitroreducens*	0	1	0
*Pseudomonas plecoglossicida*	0	1*	0
*Pseudomonas straminea*	0	1	0
*Pseudomonas tolaasii*	0	0	5*
*Pseudomonas veronii*	6*	0	0
*Sphingomonas* sp.	0	0	1
*Stenotrophomonas maltophila*	0	1	0
*Stenotrophomonas* sp.	0	0	1*

Species that showed anti-*Bd* activity are marked with an asterisk. Each cell indicates the number of individuals carrying a bacterial species. N = 8, 5, 7 toads respectively.

**Figure 2:**
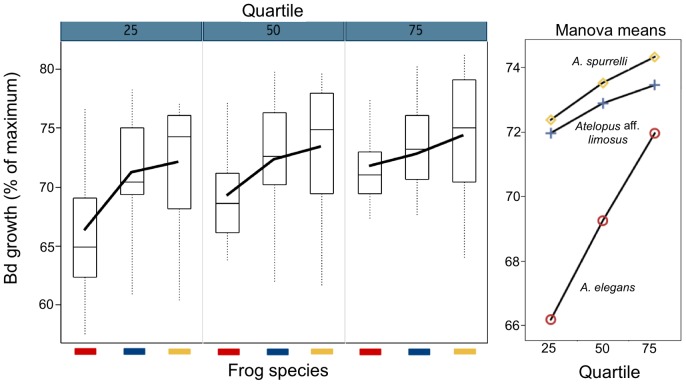
Differences in *Bd*-growth averaged from all anti-*Bd* bacterial isolates on each frog species (color code). Growth is estimated at three distances (quartiles 25, 50 and 75) from the bacteria by using spline regression models. Left: original variation as summarized by boxplots and average lines. Right: means for each frog-quartile combination as estimated from the corresponding Manova model. *Atelopus elegans* is the only species we found infected with *Bd* in its natural habitat.

**Figure 3:**
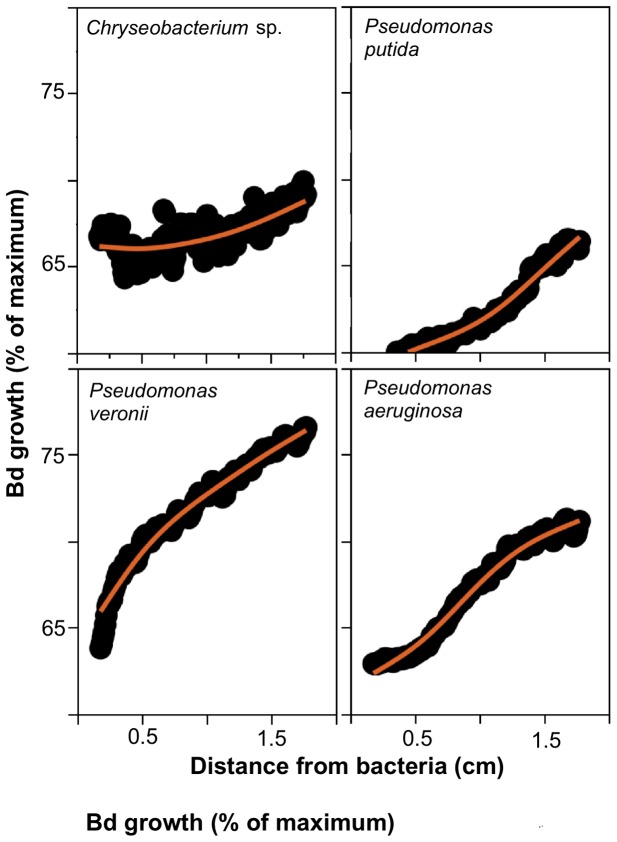
Representative examples of *Bd* growth at varying distances from putatively inhibitory bacteria. Lines denote spline regression models (Lambda  = 1.0) with R^2^ values of 0.62 (*Chryseobacterium* sp.) and above 0.95 (all the other ones).

**Figure 4:**
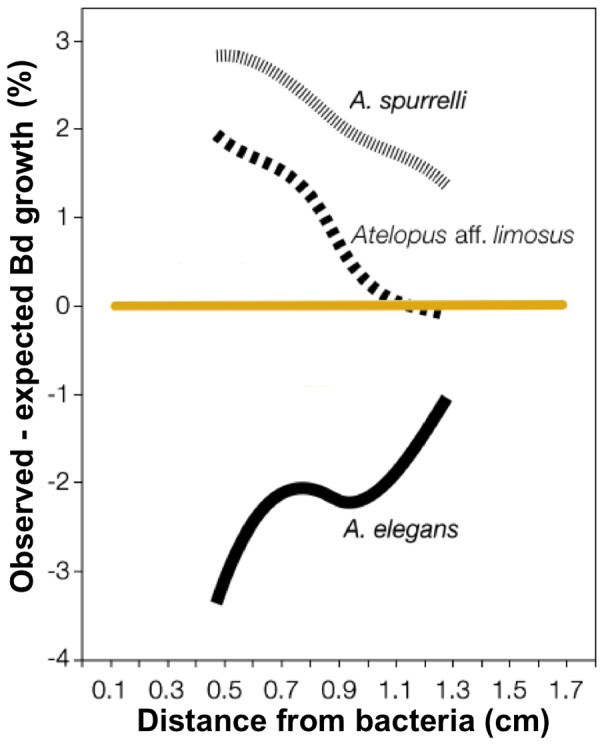
Residuals of *Bd* growth after the toad species from which the bacteria were recovered. Residual analysis of the aforementioned model to summarize differences between bacterial isolates grouped after the frog species from which they were recovered. *Atelopus elegans* is the only species we found infected with *Bd* in its natural habitat.

A generalized spline model was fitted to *Bd* growth as measured within the Petri dish at variable distances to query bacteria (Fig. 1). The regression residuals were re-analyzed by toad species and showed clearly that bacterial strains isolated from *A. elegans* caused greater *Bd* growth inhibition *in vitro* as compared to the other toad species (Fig. 2). To formally compare the results of antagonism assays, we fitted a spline model (*Bd* growth as a function of distance to the query bacteria) for each bacterial strain (Fig. 3) and interpolated *Bd* growth values at the first, second and third quartile of the growth function. Strains isolated from the skin of *A. elegans* showed higher anti-*Bd* activity (i.e. lower *Bd* growth; Manova, Frog, F = 3.292, DF  = 2, P = 0.0490) especially at the first and second quartiles of the growth curves (Manova, Frog x Quartile interaction, Wilks' lambda value  = 0.619, DF  = 4, P = 0.0024, N = 38 tests; Fig. 4).

## Discussion

Our results demonstrated that 12 bacterial species isolated from three *Atelopus* species from the Colombian lowlands inhibit *Bd* growth. The composition of anti-*Bd* bacterial communities significantly varied between toad species or perhaps localities. The strongest anti-*Bd* activity was measured in bacteria isolated from *A. elegans*, the only species that tested positive for the pathogen. Our data suggest two evolutionary mechanisms behind amphibians' resistance to chytridiomycosis, an exaptation or an adaptation against the selective pressure represented by *Bd.*

Several studies have demonstrated that the presence of bacteria with antifungal activity in amphibian skin inhibits *Bd* and prevents morbidity and mortality. The addition of bacteria with antifungal properties seems to reduce the effects of the disease on experimentally infected individuals [25], [26], [29], [30]. Our results revealed that two *Pseudomonas* (*P. tolaasii* and *P. aeruginosa*) species exhibited stronger (63 and 64% of maximum *Bd* growth as measured at the 25% quartile) anti-*Bd* activity than bacteria of any other isolated genera (69%). These results are supporting the findings of Lam et al. [47], and Walke et al. [48], which recognized that in *Rana muscosa* and *R. sierrae*, and in *Hyalinobactrachium collymbiphyllum* respectively, bacterial strains of the genus *Pseudomonas* were common residents and showed strong inhibition of *Bd* growth. Our results allow us to suggest that those bacterial species producing a larger inhibition zone may be good candidates to be used in probiotic therapies. Nevertheless, since the laboratory conditions probably differ from the host's skin, it is important to run anti-*Bd* assays using bacteria with proved efficiency for delaying or inhibiting *Bd* growth *in vitro*.

McKenzie et al. [49] suggest that skin bacteria are strongly species-specific across sites or even among species occurring in the same pond. The low number of shared bacteria found in this and other research [49], suggest that studies at the local level are important for understanding the mechanisms behind the frog-pathogen-bacteria interaction. Of course, the proportion of bacteria shared among toad species is probably underestimated since we worked only with cultivable bacteria. In any case, since bacterial communities may covary with distance or geographic conditions, it is important to look for candidate local strains with strong anti-*Bd* activity before considering bioaugmentation assays and eventual probiotic treatment of chytridiomycosis.

*Atelopus elegans*, the only species in our study that tested positive for *Bd*, holds the skin bacteria with the strongest anti-*Bd* action. Although admittedly non-replicated, the pattern suggests that the species' current bacterial community may have resulted from natural selection represented by *Bd* infection. If so, we would be witnessing a post-infection or post-decline event in *A. elegans*, where frogs and pathogen are now coexisting after a critical period of strong natural selection. Alternatively, the anti-*Bd* bacterial microbiota of the three toad species may represent an exaptation, a pre-existing (extended) phenotype that eventually subserves protection against *Bd* pathogenic infection. To discriminate between both the adaptation and exaptation scenarios, we need longitudinal (i.e. historical within the same population) data or geographic (i.e. between populations) comparisons on bacterial composition of infected and uninfected frogs. In the latter case, Woodhams et al. [26], have already showed that *Rana muscosa* from Conness in Yosemite National Park hosts a significant proportion of anti-*Bd* bacteria that inhibited *Bd* growth and persisted for six years in the presence of the chytrid fungus, whereas the Sixty Lake population was devastated by chytridiomycosis. For evolution to occur on skin microbiota, the ability to acquire or maintain certain bacteria should be heritable. Frog skins might vary in their habitability to different bacterial taxa due to their skin secretions or skin humidity; also, between species differences in habitat use may affect the probability of acquiring certain bacterial taxa. Both represent just speculative hypotheses that deserve rigorous testing.

We cannot rule out the possibility that high temperatures delay or reduce *Bd* growth in the studied *Atelopus* species, since they inhabit lowland forests of Colombian coasts with average annual temperatures around 27°C. High environmental temperatures can be directly involved with the growth control of the pathogen. Laboratory tests have shown that at temperatures higher than 25°C the pathogen either decreases zoospore production or dies [10]. Moreover, other studies showed that *Bd*-infected frogs exposed to warmer temperatures lived longer [50] and that the prevalence of infection was considerably lower [51]. In our case, high temperatures may have interacted with species-specific microbiota in allowing the survival of *A. elegans* despite the infection by *Bd*. The effect of environmental conditions in shaping the interaction between frogs and *Bd* has been already suggested [33].

Also, strain-level differences in bacteria and overall community structure may prohibit probiotics discovered on one frog species from persisting on another one. For example, *Janthinobacterium lividum*, a bacterium isolated from North American amphibians, failed to prevent or delay mortality in *Bd* exposed individuals of *Atelopus zeteki* [36]. Likewise, we cannot rule out a synergistic or detrimental anti-*Bd* effect of the whole consortium of skin microbiota, because we only evaluated the effects of individual bacteria against *Bd*. It is well known that bacterial consortia are frequently established to carry out several processes that are otherwise harder to accomplish when grown alone [52], for example the oxidation of anaerobic methane [53], [54], the metabolism of explosive compounds [55] and the enhancement of bioremediation strategies [56], among others. It has been shown that disruption in antifungal microbial communities is likely to lead to a breakdown of the protective effects of beneficial microorganisms and may lead to disease emergence [57]. Further studies are thus needed to better understand how the bacterial symbionts of frogs' skin interact among themselves and with their amphibian hosts, and how the activities performed by the skin bacteria could benefit diseased frogs.

Colombia has one of the most diverse amphibian faunas of the world with 751 described species [58], but the current conservation status of many populations and species remains unknown. This study is at the forefront of attempts to seek local bacteria involved in resistance to amphibian chytridiomycosis [59]. We found that amphibian skin microbiota is an important component of disease resistance, and moreover, we describe Neotropical bacteria that provide promising avenues for disease mitigation. In particular, probiotic therapy may be applied as a management tool to reduce the vulnerability of Neotropical amphibians to the devastating effects of *Bd* as has been demonstrated with frogs from temperate zones, where the treatment with beneficial bacteria has been shown to be highly successful to protect susceptible amphibian species from *Bd* infections [59].
